# DNA/Ag Nanoparticles as Antibacterial Agents against Gram-Negative Bacteria

**DOI:** 10.3390/nano5010284

**Published:** 2015-03-03

**Authors:** Tomomi Takeshima, Yuya Tada, Norihito Sakaguchi, Fumio Watari, Bunshi Fugetsu

**Affiliations:** 1 Graduate School of Environmental Science, Hokkaido University, Hokkaido 060-0810, Japan; E-Mail: bunshifugetsu@pari.u-tokyo.ac.jp; 2 Nissei Bio Co. Ltd., Hokkaido 061-1374, Japan; E-Mail: tada@nisseibio.co.jp; 3 Division of Materials Science and Engineering, Faculty of Engineering, Hokkaido University, Hokkaido 060-8628, Japan; E-Mail: sakaguchi@eng.hokudai.ac.jp; 4 Graduate School of Dental Medicine, Hokkaido University, Hokkaido 060-8586, Japan; E-Mail: watari@den.hokudai.ac.jp; 5 Policy Alternative Research Institute, The University of Tokyo, Tokyo 113-0032, Japan

**Keywords:** antibacterial, DNA/Ag nanoparticles, electrostatic interaction, Gram-negative bacteria, nanoparticle immobilization, salmon milt DNA

## Abstract

Silver (Ag) nanoparticles were produced using DNA extracted from salmon milt as templates. Particles spherical in shape with an average diameter smaller than 10 nm were obtained. The nanoparticles consisted of Ag as the core with an outermost thin layer of DNA. The DNA/Ag hybrid nanoparticles were immobilized over the surface of cotton based fabrics and their antibacterial efficiency was evaluated using *E. coli* as the typical Gram-negative bacteria. The antibacterial experiments were performed according to the Antibacterial Standard of Japanese Association for the Functional Evaluation of Textiles. The fabrics modified with DNA/Ag nanoparticles showed a high enough inhibitory and killing efficiency against *E. coli* at a concentration of Ag ≥ 10 ppm.

## 1. Introduction

Metal nanoparticles, due to their extraordinary physical, chemical, and biological properties together with their unique morphologies, have been the key materials epitomizing the field of nanotechnology [1,2,3,4,5]. Silver (Ag) nanoparticles typify the common metal based nanoparticles and have been already used in some commercially available products, such as refrigerators, mobile phones, clothes, plasters, toothbrushes, cosmetics, catheters, bandages, scalpels, and needles [6]. Ag nanoparticles in these particular products function mainly as antibacterial agents against bacteria, fungi and/or viruses [6,7,8,9,10,11,12]. Ag nanoparticles release free Ag(I) ions, the free Ag(I) ions are capable of interacting with the negatively charged bacterial cell wall, deactivating cellular enzymes, disrupting membrane permeability, and as a result, leading to cell lysis and cell death [13,14]. The particular antibacterial efficiency of Ag nanoparticles depends on both their size [15] and shape [16] and higher antibacterial efficiency was obtainable for particles with larger surface areas. In addition, Ag nanoparticles can inhibit biofilm formation for *E. coli*, *P. aeruginosa* and *S. proteamaculans* [17] and the possible toxicity to the environment for the Ag nanoparticles is considered to be much lower than that of the toxicity of other metals and/or organic based antibacterial agents [18,19,20].

Many methods have been developed for preparing the metal based nanoparticles. The so-called wet-chemical reduction method has been the cornerstone approach, due to its low cost, being easier to operate and its high suitability for a wide range of metal based nanoparticle preparations. Precise control of the rate of particle growth during the reduction of the precursors (metal ions being commonly used) in the presence of suitable templates is the key for metal based nanoparticle preparations via the wet-chemical reduction method. In addition, adding a desirable stabilizer to prevent the metal nanoparticles forming aggregates is also an important approach in preparing metal based nanoparticles as highly stable suspensions. Polysaccharides [21,22,23], proteins [24], and nucleic acids [25,26,27,28,29,30] have been used as the so-called biopolymer based stabilizers and metal nanoparticle suspensions of high stability are obtainable in an easier manner.

In a previous study, we reported a novel approach to large scale production of Ag nanoparticles using DNA extracted from salmon milt as both the template and stabilizer [31]. Ag nanoparticles with average diameters smaller than 10 nm and highly stable in water were obtained. The DNA/Ag nanoparticles were found to carry highly negative potentials, providing a simple yet desirable approach to immobilization of the DNA/Ag nanoparticles through electrostatic interactions for practical applications. In this study, the DNA/Ag nanoparticles were immobilized onto the surface of cotton based fabrics through electrostatic interactions and their potential as antibacterial substances was evaluated using *E. coli* as the typical Gram-negative bacteria. New insights into the mechanism of antibiotic effectiveness of the DNA/Ag/cotton-fabrics against the targeting bacteria were observed.

## 2. Results

### 2.1. Characteristics of Size Distribution and Crystal Structure of the DNA/Ag Nanoparticles

**Figure 1:**
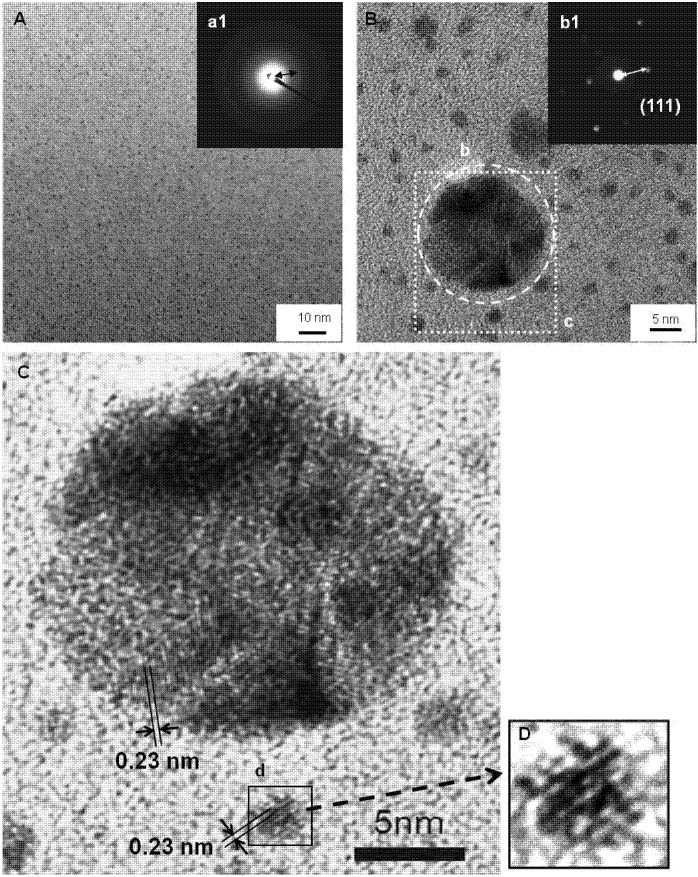
High-resolution field emission transmission electron microscope (FE-TEM) images of the DNA/Ag nanoparticles. The insets in (**A**) and (**B**) show the corresponding selected area electron diffraction pattern (a1) recorded from the whole area (**A**) and the nano-beam diffraction pattern (b1) from the area marked with the dashed line circle (b), respectively. (**C**) and (**D**) are the enlarged images from the areas indicated with the dotted square (c) in (**B**) and with the solid square (d) in (**C**), respectively.

An aqueous suspension containing Ag nanoparticles at a higher concentration (5.3 × 10^−2^ mol/L·Ag) was obtained by reducing Ag(I) ions using tetrahydroborate (NaBH_4_) as the reductant in the presence of salmon milt DNA (approximately 20,000 Da, *i.e.*, 60 nucleotides, single-stranded). Figure 1A,B shows typical images observed using field emission transmission electron microscope (FE-TEM), of particles having diameters ranging from 2 to 20 nm. The nano-beam electron diffraction pattern (Inset b1 of Figure 1B) of the particle having a size of about 20 nm (b in Figure 1B) showed a single-crystal spot pattern with other faint patterns suggesting that the larger sized particles are polycrystals. The lattice spacing was calculated as follows:

*L*λ = *dR* (1)

where *L* is the camera length, λ is the wavelength of the TEM beam, *d* is the lattice spacing and *R* is the measured diffraction ring radius or spot distance. The lattice spacing corresponding to the nearest spot was 0.24 nm, close to the (111) plane of face-centered cubic (FCC) Ag. A broad ring pattern (Inset a1 of Figure 1A) with the same diffraction ring radius as the spot in Figure 1B is observed in the selected area electron diffraction pattern from the wider area of the distributed particles (Figure 1A), indicating that the particles consist of Ag crystals. The lattice fringes with a spacing of 0.23 nm are shown in the enlarged image of the Ag nanoparticle both with a size of around 20 nm (Figure 1C) and a size of around 2 nm (d in Figure 1C,D), in good agreement with the lattice spacing calculated from the diffraction patterns.

**Figure 2:**
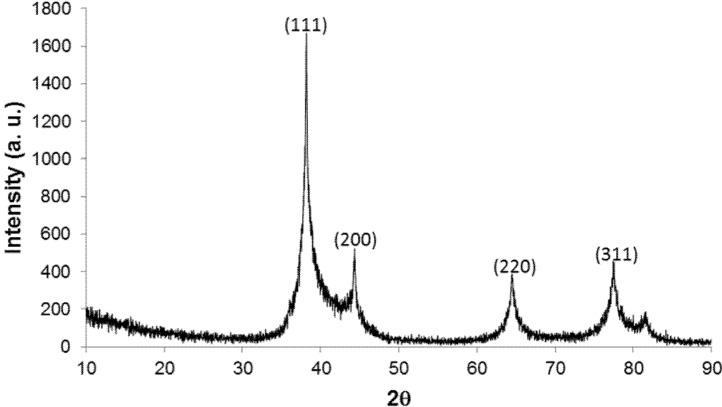
X-ray powder diffraction (XRD) patterns of the DNA/Ag nanoparticles.

X-ray powder diffraction (XRD) analysis was performed to identify the crystal phase of the DNA/Ag nanoparticles. Four peaks, each attributed to the (111), (200), (220) and (311) planes of FCC·Ag (Figure 2), respectively, were observed. The peak shape suggests a complex overlapped with a sharp and a broader peak, which indicating the existence of very fine Ag nanoparticles. Assuming that the peak attributed to the (111) consisted of sharp and broader peaks, the peak separation was done using the Lorentz function as peak shape, and the crystal size was estimated from the half-peak width of the broader peak with the equation as follows;

*D* = *K*λ/(β·cosθ) (2)

where *D* is the crystal size, *K* is a constant number, λ is the X-ray wavelength from CuKα, β is the half-peak width after correction of the equipment error and θ is the Bragg angle of the diffracted peak. The crystal size was estimated to be less than 10 nm, which was very consistent with the size as indicated from the FE-TEM images (Figure 1).

### 2.2. Use of the DNA/Ag Nanoparticles as Antibacterial Materials

#### 2.2.1. Evaluation of the Antibacterial Efficiency of the DNA/Ag Nanoparticles

**Figure 3:**
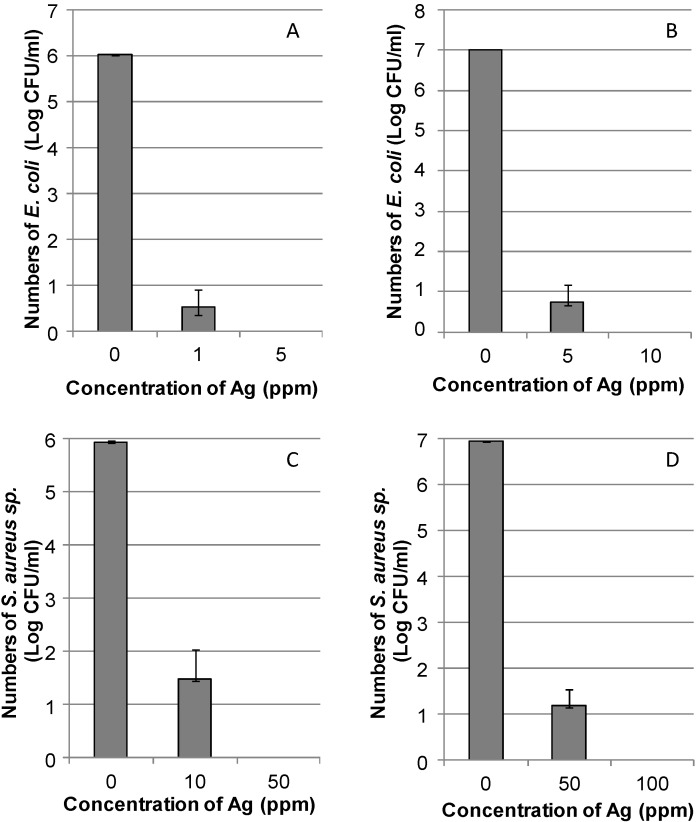
Number of colonies after incubation with a DNA/Ag nanoparticle aqueous suspension at different concentrations. (**A**) 1.04 × 10^6^ colony-forming units (CFU)/mL; (**B**) 1.04 × 10^7^ CFU/mL, of *E. coli*; (**C**) 8.65 × 10^5^ CFU/mL; (**D**) 8.65 × 10^6^ CFU/mL, of *S. aureus* were applied (without DNA/Ag nanoparticles, *n* = 2; with DNA/Ag nanoparticles, *n* = 4).

Antibacterial efficiency of the DNA/Ag nanoparticles was evaluated by using both Gram-positive *S. aureus* and Gram-negative *E. coli*. Figure 3 shows the number of bacterial colonies on nutrient agar plates after incubation with and without the DNA/Ag nanoparticles. The growth inhibition effect was observed against both kinds of bacteria in a concentration-dependent manner. For the 10^6^ and 10^7^ colony-forming units (CFU)/mL of bacteria, the concentrations of DNA/Ag nanoparticles to completely prevent bacterial growth were 5 and 10 ppm, respectively, against *E. coli* (Figure 3A,B). The concentrations found were 50 and 100 ppm, respectively, to completely inhibit against *S. aureus* (Figure 3C,D).

#### 2.2.2. Preparation of the DNA/Ag Nanoparticle Immobilized Fabrics

**Figure 4:**
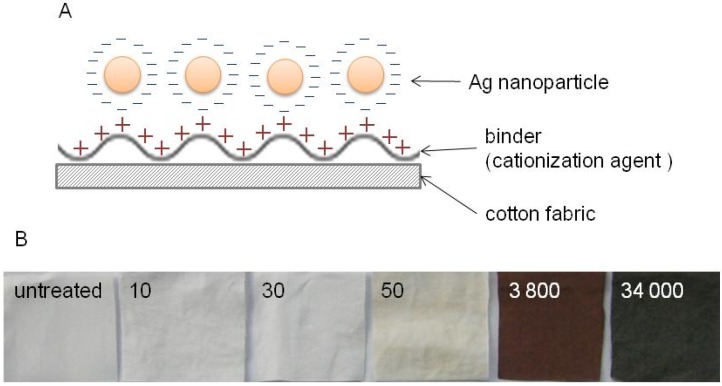
(**A**) Schematic description of the procedure for immobilizing DNA/Ag nanoparticles on to the cotton fabric; (**B**) Photographs of the DNA/Ag nanoparticle immobilized fabric samples. The amounts of immobilized Ag nanoparticles are shown as Ag (ppm).

The DNA/Ag nanoparticles were immobilized on the surfaces of cotton fabrics based on electrostatic interaction (Figure 4A). The cotton fabric samples were first modified with a cationic polymer, consisting of diallyl(3-chloro-2-hydroxypropyl)amine hydrochloride-diallyldimethylammonium chloride copolymer. The amount of immobilized Ag on the fabric was estimated by using inductively coupled plasma atomic emission spectrometry (ICP-AES). Cotton fabrics with DNA/Ag nanoparticles at concentrations such as Ag at 10, 30, 50, 3800 and 34,000 ppm were prepared (Figure 4B).

#### 2.2.3. Ag(I) Ions Release Ratio from the DNA/Ag Nanoparticle Immobilized Fabrics

**Figure 5:**
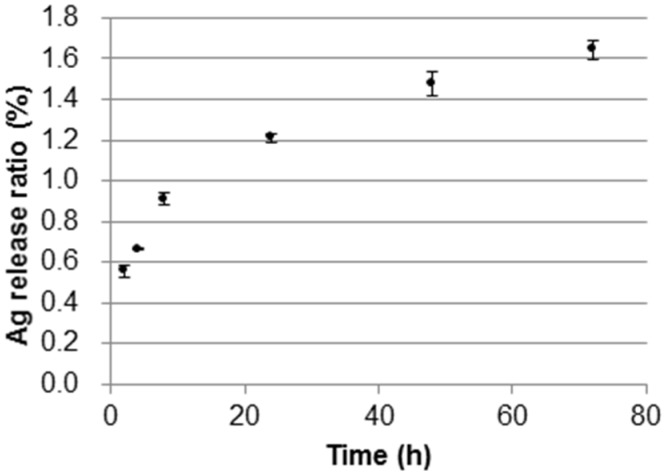
Ag(I) ions release profiles for the DNA/Ag nanoparticle immobilized cotton fabric (34,000 ppm as Ag). Error bars represent maximum and minimum value (*n* = 2).

Ag(I) ions release behavior was tested for the fabric with the immobilized DNA/Ag nanoparticles of 34,000 ppm as Ag. The fabric sample (approximately 200 mg) was immersed in a 50 mL glass vial containing 20 g water and then placed under shaking at 37 °C. The typical peak for Ag nanoparticles at around 410 nm was not observed in the UV-Vis absorption spectrum of the collected water samples, indicating a fact that the DNA/Ag nanoparticles had been firmly immobilized with the cotton fabrics. The water samples were analyzed using ICP-AES and Ag(I) ions were identified. Concentrations of Ag(I) ions in the water samples were found to be 1.9 ppm after 1 h and 6.4 ppm after 72 h of the release test, respectively. Ag(I) ion release ratio (Figure 5) was calculated as follows:

*Q* = *N*_a_/*N*_o_ × 100 (3)

where *Q* is the release ratio, *N*_a_ is the Ag(I) ion content in the water sample at each time point of sample collecting, and *N*_o_ is the amount of the total immobilized Ag on the fabric (approximately 6.8 mg per sample). The amount of Ag(I) ions released from the fabric was found to be at around 1% and 2%, after 8 h and 72 h of the testing, respectively. The increase in the release ratio is predicted to reach a constant value according to Fick’s law of diffusion.

#### 2.2.4. Evaluation of the Antibacterial Efficiency of the DNA/Ag Nanoparticle Immobilized Fabrics

**Figure 6:**
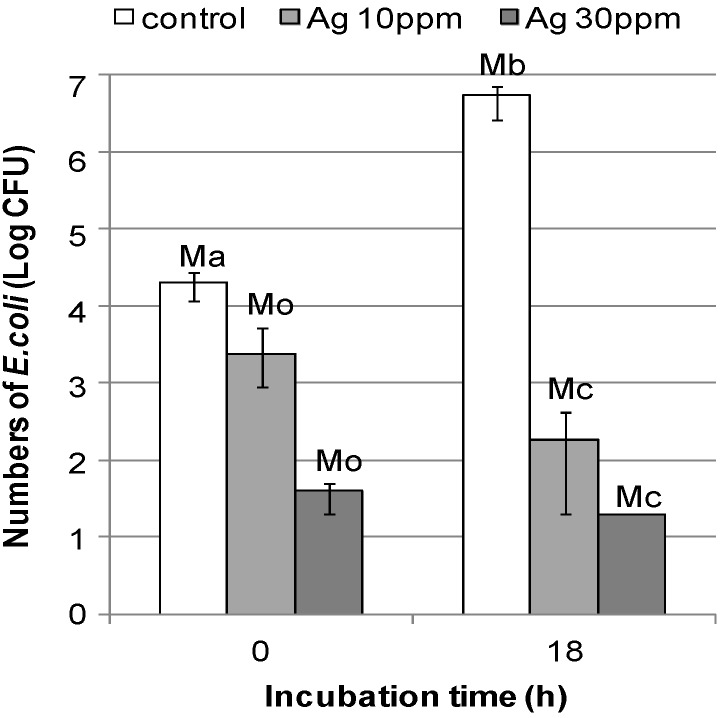
Numbers of colonies found for the untreated fabrics (controls) and for the DNA/Ag nanoparticles immobilized fabrics (10 and 30 ppm as Ag) at the starting point (0 h) and after 18 h incubation (18 h). If no colonies were observed, the number was regarded as less than 20 CFU/mL and was shown as 20 CFU/mL Error bars represent maximum and minimum value (*n* = 3).

Antimicrobial efficiencies of the DNA/Ag nanoparticle immobilized fabrics were evaluated according to the Japanese Industrial Standard (JIS L 1902/2008) [32] using *E. coli*; the initial concentration of the bacteria seeding was chosen at 2.6 × 10^5^ CFU/mL. Numbers of the bacteria at the beginning and at the time after incubation for 18 h are shown in Figure 6. For the controls (samples without DNA/Ag nanoparticles), the numbers of the bacteria increased to 5.40 × 10^6^ after 18 h of incubation. In contrast to the controls, the number of bacteria inoculated on the DNA/Ag nanoparticle immobilized fabrics with 10 ppm Ag decreased to 1.82 × 10^2^; for the fabrics with 30 ppm Ag, colonies were not observed after 18 h incubation.

Values of bacteria growth activity (BG), bacteriostatic value (BS) and bactericidal value (BC), were calculated; Table 1 summarizes the data. As denoted in the Antibacterial Standard of Japanese Association for the Functional Evaluation of Textiles (JAFET), when the BS value is found to be larger than 2.2, the test materials are classified as materials considerably capable of inhibiting bacteria; when the BC value is found to be greater than 0, the test materials are materials considerably capable of killing bacteria [33]. In this study, both the DNA/Ag nanoparticle immobilized fabrics (10 and 30 ppm·Ag) provided a BS value > 2.2 and a BC value > 0, indicating that their bacteria inhibitory efficiency and bacteria-killing effectiveness are high enough as antibacterial materials.

**Table 1:** Antibacterial evaluation values.

Testing Item	Test Results (Colony Numbers)			Reference [33]
	Controls	DNA/Ag Nanoparticle Immobilized Fabrics		
		10 ppm	30 ppm	
Ma	2.05 × 10^4^	-	-	
Mb	5.40 × 10^6^	-	-	
Mo	-	2.35 × 10^3^	<40	
Mc	-	1.82 × 10^2^	<20	
BG	2.4	-	-	
BS		>3.6	>2.7	>2.2 Inhibitory effect
BC		>2.3	>3.0	>0 Killing effect

Notes: Ma, Mb, Mo, Mc: corresponds to Figure 6; BG: the values of bacteria growth activity = log(Mb/Ma); BS: bacteriostatic value = log(Mb/Ma) − log(Mc/Mo); BC: bactericidal value = log(Ma/Mc); The mean value was used as the number of the bacteria (*n* = 3). These values were calculated according to JIS L 1902/2008 [32]. If no colonies appeared, the number was regarded as less than 20 CFU/mL.

## 3. Discussion

### 3.1. Formation Mechanism of DNA/Ag Nanoparticles

The zeta-potential of the DNA/Ag nanoparticles in water was found to be −73.8 mV, which was the value identical to the zeta-potential for the entire DNA. This fact suggests that the surfaces of the Ag nanoparticles were covered with DNA [31]. The molar ratio of nucleotide to Ag of the DNA/Ag nanoparticle was calculated to be 1:18 from the weight ratio of 14.3/85.2, measured using ICP-AES [31]. The number of Ag atoms combined to one molecule of the 60 nucleotides based DNA was calculated to be around 1080. The size of the Ag core, consisting of this number of Ag atoms covered with one molecule of the 60 nucleotides based DNA, was calculated to be 3.7 nm, which was in good agreement with the images in Figure 1 as well as the estimated crystal size calculated from the peak attributed to (111) in the XRD spectra.

**Figure 7:**
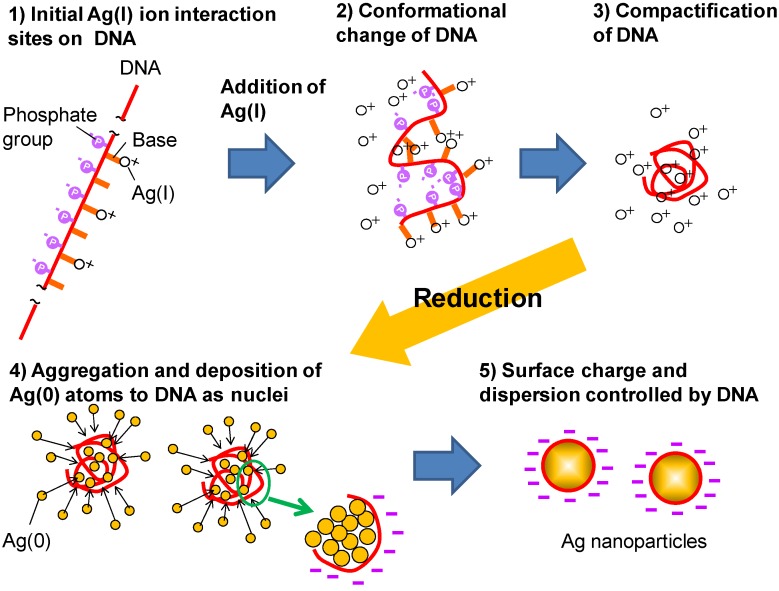
Proposed formation mechanism of the DNA/Ag nanoparticles.

Figure 7 shows a schematic image for describing a possible mechanism of the DNA/Ag nanoparticles formation: (i) A certain number of the free Ag(I) ions interacted with the nucleotide base of DNA to form a Ag(I)/DNA complex; (ii) The Ag(I)/DNA complex was densely packed with a certain number of the free Ag(I) ions being caged within this restricted range; (iii) Once NaBH_4_, the reductant was introduced, these caged Ag(I) ions were reduced to Ag(0) atoms and this could be seen as the Ag core of the nanoparticles; (iv) DNA attached on to the core Ag(0) nanoparticles and formied the high natively charged DNA/Ag nano-sized hybrids.

### 3.2. Antibacterial Effect of the DNA/Ag Nanoparticles

Inhibitory concentrations of Ag nanoparticles reported in previous articles were in the range of 0.5–20 ppm against *E. coli* and 7.5–33.7 ppm against *S. aureus* for 10^3^–10^5^ CFU/mL [6], respectively. In this study, the inhibitory concentrations of the DNA/Ag nanoparticles were 5 ppm against *E. coli* and 50 ppm against *S. aureus* for the bacteria at 10^6^ CFU/mL. In other words, the antibacterial efficiency of the DNA/Ag nanoparticles prepared in this study was found to be comparable or even better than that of other types of Ag nanoparticles reported in previous studies. In addition, the DNA/Ag nanoparticles showed higher antibacterial efficiency against *E. coli*, compared with *S. aureus*. Gram-positive bacteria are unique to the Gram-negative bacteria; the cell wall of Gram-positive bacteria is about a 30 nm thick peptidoglycan layer, while for the Gram-negative bacteria it is about 2–3 nm [8]. Feng *et al.* [13] reported that Ag ions are capable of stopping the DNA replication function and/or making proteins become inactivated; morphological changes of *S. aureus* after the treatment with Ag ions were found to be smaller than that for *E. coli*. Sondi *et al.* [4] reported that after the treatment with Ag nanoparticles, *E. coli* cells were damaged and a so-called formation of “pits” in the cell wall of the bacteria was observed. Ag nanoparticle accumulation on to the bacterial membrane leads to a significant increase in permeability and thereby is the cause of cell death [4], although so-called free-radical generation caused by the free Ag(I) ions is the widely accepted explanation for understanding the mechanism of growth-inhibition by Ag nanoparticles [5].

For the DNA/Ag nanoparticles, free Ag(I) ions released from the core Ag nanoparticle also play important roles in determining antibacterial efficiency. The DNA/Ag nanoparticles carry highly negative charges; this can lead to preventing microorganisms from contacting the Ag nanoparticle surface. In other words, the DNA/Ag nanoparticles function mainly as nano-sized reservoirs providing free Ag(I) ions in a continuous manner in to the media. The free Ag(I) ions, once reached to the bacteria function as antibacterial reagent in the manner similar as the mechanism proposed for the Ag(I) free ions provided by using other kinds of Ag nanoparticles as the providers.

## 4. Experimental Section

### 4.1. Materials

DNA was obtained from Nisseibio Co. Ltd. (Eniwa, Japan). The molecular weight of the DNA was estimated by HPLC, and the determination of double-stranded and/or single stranded of DNA was made by measuring the increase in absorbance at 260 nm upon heating the DNA solution at 95 °C. Silver nitrate (AgNO_3_ special grade), potassium dihydrogen phosphate (KH_2_PO_4_, special grade) and polyoxyethylene sorbitan mono-oleate (Tween 80^®^) were purchased from Kishida Chemical Co. Ltd. (Osaka, Japan). Ammonia solution (10%) and sodium borohydride (NaBH_4_ chemical grade) were purchased from Wako Pure Chemicals Industries Ltd. (Osaka, Japan). Potassium peroxydisulfate (for N and P analysis grade) and sodium carbonate (Na_2_CO_3_, special grade) were purchased from Kanto Chemical Co. Inc. (Tokyo, Japan). Sodium hydroxide (ACS grade) was purchased from Sigma-Aldrich (St. Louis, MO, USA). Ultra-pure water (supplied from Milli-Q water purification systems, Merck Millipore, Billerica, MA, USA, >18 MΩ) was used in all experiments.

*S. aureus* (AHU1142) was obtained from the Faculty of Agriculture, Hokkaido University (Sapporo, Japan). *E. coli* (NBRC3301) was purchased from the National Institute of Technology and Evaluation (Chiba, Japan). Peptone (Bacto Peotone) and beef extract (desiccated), which were purchased from BD (Franklin Lakes, NJ, USA), Agar (BA-10, high quality) from Ina Food Industry Co. Ltd., (Ina, Japan), were used to prepare the culture medium.

Cotton broad fabric, fabric mass of 122.5/m^2^, was purchased from Shikisensya (Osaka, Japan). Cationic polymer solution (DANSHADE^®^) consists of diallyl(3-chloro-2-hydroxypropyl)amine hydrochloride-diallyldimethylammonium chloride copolymer which was used as a cationic agent to make the cotton fabric, carrying positive charges, was provided from Nittobo Medical (Tokyo, Japan).

### 4.2. Preparation and Characterization of the DNA/Ag Nanoparticles

The DNA/Ag nanoparticles were prepared as described in previous report [31]. An aqueous AgNO_3_ (10^−1^ mol/L) was mixed with a DNA solution (10^−2^ mol/L nucleotide in 1% aqueous ammonia) at a volume ratio of 2:1, then a NaBH_4_ solution (1 mol/L in 10% aqueous ammonia) was added at one tenth of the volume of the aqueous AgNO_3_ to the mixture as a reductant at around 0 °C. The supernatant, which contained the DNA/Ag nanoparticles, was dialyzed using a dialysis membrane (molecular weight cut-off: 14,000, Viskase Companies Inc., Darien, IL, USA) in water for approximately 24 h. A detailed observation of the DNA/Ag nanoparticles was performed by high resolution observation using FE-TEM (JEM-2010F, JEOL, Akishima, Japan). The crystal structure and size of the DNA/Ag nanoparticles were analyzed by XRD measurement with an X-ray diffractometer (RINT 2000/PC, Rigaku Corporation, Akishima, Japan) with CuKα radiation operated at 40 kV and 40 mA.

### 4.3. Evaluation of the DNA/Ag Nanoparticles as Antibacterial Materials

#### 4.3.1. Antibacterial Evaluation of the DNA/Ag Nanoparticles

Antibacterial efficiency of the DNA/Ag nanoparticles was examined against *S. aureus*, AHU1142, as Gram-positive bacteria, and *E. coli*, NBRC3301, as Gram-negative bacteria. The DNA/Ag nanoparticle aqueous dispersion was sterilized by filtration through a 0.45 μm syringe filter (Minisart, Sartorius Stedim Biotech, Goettingen, Germany), and then diluted (2–200 ppm as Ag) with phosphate buffer solution (0.25 M KH_2_PO_4_, pH 7.2). The bacteria were pre-cultured in nutrient broth (5% peptone, 3% beef extract) for 16 h at 37 °C. The culture solutions were diluted with phosphate buffer solution (0.25 M KH_2_PO_4_, pH 7.2), and mixed with the DNA/Ag nanoparticle dispersion at a volume ratio of 1:1. The mixture (0.1 mL) was put into bacterial petri dish (ϕ 90 mm) with 20 mL of nutrient agar medium (5% peptone, 3% beef extract, 15% agar), and incubated for 24–48 h at 37 °C.

#### 4.3.2. Preparation of the DNA/Ag Nanoparticle Immobilized Fabrics

Cotton fabric was treated with the cationic agent diluted with water (1–10,000 ppm) for 20 min at 80 °C by using a hot plate stirrer, and then Na_2_CO_3_ was added to make the solution up to 1 wt.% Na_2_CO_3_. The mixture was kept for 30 min at 80 °C. The fabric was washed with deionized water and was then soaked in the DNA/Ag nanoparticles dispersion (5–5900 ppm as Ag) for 30 min. After careful washing with deionized water, the fabric was dried in air. The amount of the immobilized Ag was measured using ICP-AES (ICPE-9000, Shimadzu, Kyoto, Japan) after hydrolysis in the presence of aqueous potassium peroxydisulfate (4.0%) at 120 °C.

#### 4.3.3. Ag(I) Ions Release from the DNA/Ag Nanoparticle Immobilized Fabrics

A small piece (approximately 0.2 g) of the DNA/Ag nanoparticles immobilized fabric (34,000 ppm as Ag) was immersed into water (20 mL) in 50 mL glass vial container with screw cap. The container was shaken at 37 °C in a swing shaker (around 110 rpm). The water (1 mL) was sampled at 2, 4, 8, 12, 24, and 48 h, and the Ag concentrations were measured with ICP-AES.

#### 4.3.4. Antibacterial Evaluation of the DNA/Ag Nanoparticle Immobilized Fabrics

Antibacterial assay of the DNA/Ag nanoparticle immobilized fabrics (with 10 and 30 ppm as Ag) was performed according to JIS L 1902/2008—Adsorption test method [32] using *E. coli*. as follows: the bacteria were pre-cultured in nutrient broth for 16 h at 37 °C, and were then diluted with 20-fold diluted nutrient broth (pH 6.8) as bacteria suspension containing 1–3 × 10^5^ CFU/mL. The bacteria suspension (0.2 mL) was inoculated to each specimen (0.4 g). Three untreated specimens and test specimens were washed immediately with 0.2% Tween 80-containing saline (20 mL), and the washed bacterial suspension (1 mL) was taken into a bacterial petri dish (ϕ 90 mm) with 20 mL of nutrient agar medium and then incubated at 37 °C for 24–48 h to measure the number of bacteria. The other three untreated specimens and the treated specimens were incubated at 37 °C for 18 h, and the number of bacteria was determined in the same way as at the beginning of the contacting experiment. The values of BG, BS and BC of the DNA/Ag nanoparticle immobilized fabrics were then calculated according to JIS L 1902/2008 [32].

## 5. Conclusions

DNA modified Ag nanoparticles with average diameters smaller than 10 nm were successfully produced by using natural DNA extracted from salmon milt as the template. The resultant DNA/Ag nanoparticles carry highly negative charges; this provides a desirable approach to immobilization of Ag nanoparticles onto fabric surfaces via electrostatic interactions. Cotton based fabrics modified with the DNA/Ag nanoparticles showed a sufficiently high antibacterial efficiency against *E. coli*.
